# Assessing the Impact of Behavioral Sciences Interventions on Chronic Disease Prevention and Management: A Systematic Review of Randomized Controlled Trials

**DOI:** 10.3390/ijerph21070837

**Published:** 2024-06-27

**Authors:** Rafael Corrêa, Matheus Britto Froner, Benjamin Miranda Tabak

**Affiliations:** School of Public Policy and Government, Getulio Vargas Foundation, SGAN 602 Módulos A,B,C, Asa Norte, Brasília 70830-020, Brazil; froner2433@gmail.com (M.B.F.); benjamin.tabak@fgv.br (B.M.T.)

**Keywords:** behavioral sciences, chronic disease, self-management, prevention, public health

## Abstract

Studies have highlighted the importance of applying Behavioral Sciences interventions to develop equity in the prevention of chronic diseases in the public health domain. Our study aims to assess the evidence of this influence. We undertook a systematic review study using the electronic databases PubMed, Web of Science, Scopus and Cochrane, searching for work published between 2013 and 2023. The research analyzed the influence of Behavioral Sciences intervention studies on public health. This review was registered and published in PROSPERO, registration number CRD42023412377. The systematic search identified 2951 articles. The review analyzed 26 studies. The quality assessment of the articles showed an overall average of 74%, with the majority of studies being of high quality. The interventions with the best evidence for chronic diseases used framing messages, nudges and vouchers. Messages with incentives also showed satisfactory evidence. The most prevalent outcomes were related to screening tests and patient adherence to treatment. The current state of decision-making remains mainly at the patient level, with potential for further exploration of the roles of healthcare professionals and decision-makers in future research efforts. Limitations relate to the heterogeneity of the study sample, which hinders a more precise analysis of specific interventions and outcomes in chronic diseases.

## 1. Introduction

The traditional economic theory applied in public health assumes that agents act rationally, utilizing all available information to select options to maximize their well-being or satisfaction [1]. This hypothesis serves as the bedrock for predicting human behavior across many fields. However, insights stemming from the discipline of Behavioral Sciences, enriched by psychological research, reveal that this rationality is often constrained [2]. The decision-making agent is hindered by various cognitive biases, resulting in choices that do not necessarily lead to optimal well-being or happiness [3].

From a Behavioral Sciences standpoint, deviations from rationality are not anomalies but ordinary and anticipated aspects of human behavior. These expected deviations are particularly salient when individuals make health-related decisions. Such lapses in rational judgment can lead to negligence regarding personal health care and a failure to appraise all available information when making critical choices. Consequently, a burgeoning body of literature explores the far-reaching implications of Behavioral sciences within the health field, especially in medicine [4].

Studies in the health field using standardized interventions based on the rational decision-making approach rely on education, financial incentives, and the regulation of healthcare quality [5]. Behavioral Sciences-based interventions involve framing messages, nudges and vouchers modifying individuals’ choice architecture. Thus, this design and strategy can be used effectively in different health systems [6,7].

Nudges change how options are presented, making it easier to choose the best one. In a nudge, you do not force the decision-maker to do something; you show them choices so that they can make a choice that might be more appropriate for them. The decision-maker is free to choose [8]. In experiments, to incentivize participants, one can give monetary rewards, when money is provided directly to the participants, or vouchers, which would be rights, coupons, or tickets, which participants can exchange for goods or services, or even cash after a certain period of time.

In this paper, we enhance the existing discourse by conducting a comprehensive and systematic review of scholarly articles that explore the intricate relationship between behavioral sciences and chronic diseases. Our focus is on various behaviorally informed interventions designed to address the issues arising from deviations from rational decision-making. Our research question is to what extent do Behavioral Sciences influence the development and management of chronic diseases? We provide a comprehensive examination in this paper.

Our research provides a pivotal contribution to the conversation surrounding preventive medicine. By unearthing and synthesizing the most persuasive evidence, we offer actionable strategies to refine the healthcare decision-making process. Discussions on bolstering medication adherence, implementing crucial screenings [9,10], and other meaningful deliberations necessitate meticulous analysis. Through our thorough investigation, we endeavor to highlight these critical matters and present practical solutions, thereby fostering a more nuanced understanding of how Behavioral Sciences can be leveraged to improve overall health outcomes.

## 2. Materials and Methods

This systematic review followed the PRISMA 2020 Statement protocol guidelines [11] and was registered and published at PROSPERO (http://www.crd.yourk.ac.uk/prospero/), accessed on 20 February 2024, registration number: CRD42023412377 (Table S1).

Eligibility criteria for the inclusion of studies were defined based on study design, intervention, outcomes, and language. We included Randomized Controlled Trial studies of Behavioral Sciences interventions in public health, published in English, Spanish and Portuguese from 2013 to January 2023.

The search was carried out on 27 March 2024 in the following indexed electronic databases—Cochrane, PubMed, Web of Science and Scopus—seeking to explore the field of behavioral economics and public health, using the following strategy: (Public Health [MeSH Terms]) AND (behavioral economics (BE) [MeSH Terms]). Considering that the search strategy was broad and concerned intersectoral fields of research and action, the authors initially chose to identify general studies, but due to the number of studies selected for the final sample (*n* = 85), priority themes emerged, facilitating analysis and discussion in specific areas. For this article, we will analyze intervention studies in Behavioral Sciences and the effect on chronic diseases in public health.

The screening and selection of texts were carried out with the support of the Rayyan program [12], using the PICOS strategy defined in the study protocol. The selection by titles and abstracts was carried out independently by two authors seeking consensus. However, inconsistency in decisions was assisted by a third author. The full texts were analyzed in the final sample.

Data were extracted by two independent authors and analyzed by a third author. If there was a difference between the other two, the third person resolved it. Differences were analyzed and resolved by consensus between the authors. The data considered in the extraction were author, year and country, study design, sample, instruments, study quality, interventions and outcomes.

We performed the quality assessment of the included studies using the Evidence-Based Librarianship (EBL) Critical Appraisal Checklist [13]. The tool consists of a checklist using a quantitative analysis of the articles in the systematic review to assess risk of bias. A validity analysis takes place through subclasses such as population, data collection, study design, results and ‘overall’. Raw scores are obtained from the “Yes” answers, which score 1, and the “No” and “Unclear” answers, which score 0. The answers are divided by the total number of items in each subclass as well as the total score, and multiplied by 100 to arrive at the percentage score. N/A answers are annulled and do not count as an item when calculating the percentage of subclasses and overall validity. The result is obtained from the scores “Yes” ≥ 75% and “No/Unclear” ≤ 25%, defined by the author [13].

## 3. Results

**Figure 1:**
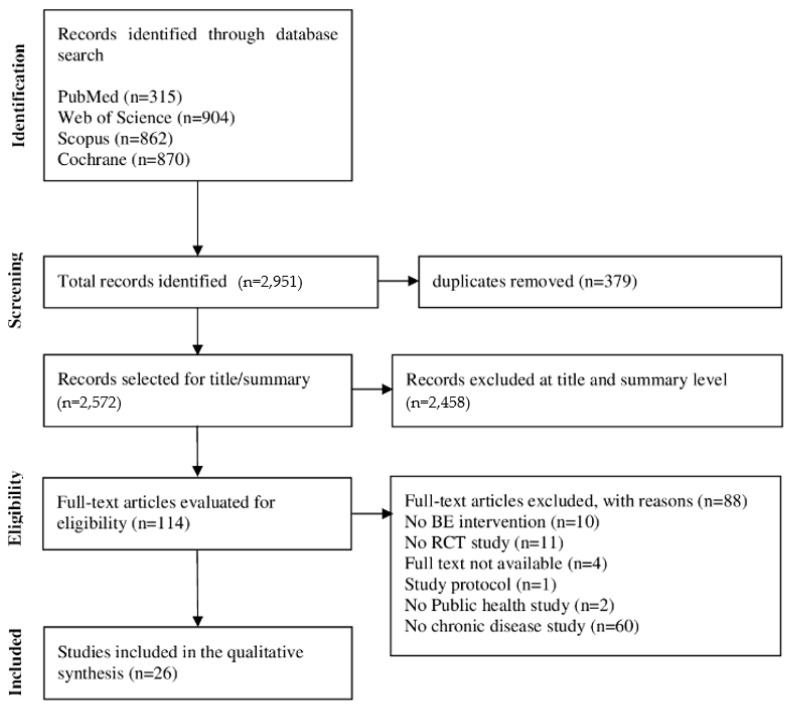
Study flowchart.

The systematic search of the databases identified 2951 articles, and excluded 379 duplicates and removed 2458 articles after analyzing titles and abstracts that did not meet the PICOS strategy described in the study protocol. Due to the use of a broad search strategy in the databases and the use of Randomized Controlled Trials (RCTs), the number of exclusions was significant at this stage of the screening. A sample of 114 articles was identified for full reading, and 88 articles were excluded. The flowchart for this systematic review is shown in Figure 1.

Interventions were based in framing messages (*n* = 13) [1,4,10,14,15,16,17,18,19,20,21,22,23] in most studies, followed by messaging and incentives (*n* = 4) [24,25,26,27], financial and mixed incentives (*n* = 4) [2,28,29,30], nudge (*n* = 3) [31,32,33] and voucher (*n* = 2) [3,9].

Regarding the results, the interventions using framing messages demonstrated results such as reduced knowledge gaps in relation to COVID-19 [14], the influence of information such as the effectiveness and cost of treatment on the increase in search intention for mammography screening exams [20], screening for prostate examination in elderly men and overtreatment of diabetes in the elderly, thus burdening the health system [19]. The practice of protected sex is related to safer decision-making time [22], increased adherence to cervical cancer screening [1], increased contraceptive treatment adherence [14], increased adherence to preventive behaviors against COVID-19 [23], an influence on physicians’ behavior and patients’ adherence to medication [4], increased active choice in colonoscopy examination [10], increased positive belief in antibiotic action in influenza [15] and uptake of HIV testing impacting on reduced transmission [16].

The studies that used messages and incentives demonstrated results such as Increased the likelihood of patients’ adherence to medical appointments [24], increased adherence to colonoscopy [25], increased mammograms received [26], and decreased blood pressure (BP) in the short term [27]. 

However, interventions that used financial and mixed demonstrating results including a reduction in the cholesterol level of patients [28], influence on physicians’ behavior and patients’ medication adherence [29], increased adherence to medicalization and decreased rehospitalization [2] and increased adherence to treatment for eye patients [30].

The interventions that used nudge demonstrated results such as Increased cervical–rectal cancer (CRC) screening [31] and contraceptive treatment adherence [32]. Finally, the voucher strategy presented results of increased the intention to adhere to screening for mammography [9] and the prioritization of patients concerning health investments by regulators [3] (Table 1).

Most studies were conducted in the United States of America (*n* = 13) [2,10,15,16,17,19,22,23,25,26,28,29,33], Singapore (*n* = 2) [9,27], Japan (*n* = 2) [24,31], the UK (*n* = 2) [1,4], China (*n* = 2) [27,30], Chile (*n* = 1) [20], Indonesia (*n* = 1) [21], Ethiopia (*n* = 1) [32], South Africa (*n* = 1) [14] and Switzerland (*n* = 1) [3] were other countries of origin.

The Randomized Controlled Trial studies included used simple RCT (*n* = 18) [1,3,4,9,10,15,16,17,18,20,21,22,23,24,25,26,27,33], clusters (*n* = 5) [19,28,29,30,32], pilot studies (*n* = 2) [2,14] and prospective studies (*n* = 1) [31].

Regarding the characteristics of the participants in the studies, most were conducted with patients (*n* = 13) [1,2,9,10,14,16,18,20,24,26,27,31,32], adults (*n* = 4) [3,4,17,23], primary care physicians and patients (*n* = 3) [15,28,29], students [22,30], primary care physicians [19], decision-makers [33], employees [25] and community health workers and patients [21] (Table S2).

The quality assessment of the articles showed an overall mean of 74%, with most studies (*n* = 14) having high quality, ≥75%, according to the EBL assessment (Table 2) [13]. Interventions using framing messages had an average of 76% overall validity; messages and incentives presented an average of 74% in the overall validity of the studies; financial and mixed incentives had an overall validity of 66% on average; nudge presented an average of 75% in the overall validity of the studies and voucher strategy presented an average of 80% in the overall validity of the studies.

## 4. Discussion

The Behavioral Sciences interventions that showed the best evidence for chronic disease used framing messages [1,4,10,14,15,16,17,18,19,20,21,22,23], nudge [31,32,33] and vouchers [3,9]. Studies in the Behavioral Sciences have shown the influence of interventions in optimizing choice and decision-making processes in public health, especially related to increased adherence to screening tests and treatment for chronic diseases.

Message framing demonstrates two characteristics, with loss-related messages generally being used more in clinical diagnostic tests, while gain-related messages are used more in the field of disease prevention [1]; consequently, framing messages influence increasing treatment adherence in public health. However, the results of the studies in this systematic review show that messages still have little evidence in studies involving public health policy-makers, who are not aware of the importance of choice architecture in health behaviors and in changing the understanding of health service delivery systems for patients with chronic diseases [34], which is one of the challenges for future research.

The most prevalent findings were related to screening examinations [1,9,10,19,20,25,26,31]. Studies show that most screenings have limited benefits for patients tested regularly, and the most common problems pointed out are “forgetfulness” and “procrastination”; strategies involving reminders by letter and telephone have shown better patient compliance with tests. However, even with high efficacy, this methodology tends to be more expensive and challenging to extend to the general population, thus burdening the public health system [35].

Patient adherence to treatment [2,18,21,24,28,29,32] was the second outcome in the study results. In many public health systems, treatment adherence is centered on the role played by the doctor, which justifies the future integration of this intervention together with other health professionals as well as patients. This approach seems advantageous for providing more significant support for treatment adherence, considering the scarcity of evidence for changing healthier habits after the intervention, thus impacting a more permanent change in patients’ health and the greater involvement of health teams [36].

To elucidate the issue of health team involvement and permanent changes in patients’ habits, an intervention study demonstrated that patient orientation, medication management with monitoring by a health professional, cognitive–behavioral intervention, messages with reminders to take medication and incentives were indicated as strategies to promote adherence to treatment [37].

Messages and incentives also presented satisfactory evidence [24,25,26,27]. Most of the results were from screening tests, were followed using financial incentives and were mixed, in the order of validity of the evidence, showing results in patients who had cardiovascular disease [24,27]. For both cases, messages and incentives usually showed a more significant result when the information was simple and objective, and when the incentives were released in the short and medium term [24,27], These considerations demonstrate an additional challenge for the process of translating knowledge into public health policies, considering that they are planned, executed and evaluated over a long period and designed for larger populations.

The studies were primarily conducted with patients [1,2,9,10,14,16,18,20,24,26,27,31,32], which leads to a reflection on the decision-making process only at the individual level. Interventions carried out with primary care physicians, workers and patients [15,28,29] are more likely to bring about collective change, but only one study was carried out with decision-makers in the health system [33], which would have a more structural impact on public health policy-making with equity. Co-produced health interventions would allow professionals, researchers and managers to develop a shared understanding that would improve the evaluation of co-produced public health actions, programs and policies applied to chronic diseases [38].

Increasing patients’ knowledge or active choices is only one step towards affecting disease behavior and exposure. We leave some reflections from the results of this systematic review, including whether other health professionals would have the same influence on patients’ adherence to chronic disease treatment? Or a change in lifestyle habits? Or again, what would be the evidence from Behavioral Sciences to show the path from awareness to impact on decision-making in health managers towards equity?

This systematic review presents some limitations related to the heterogeneity of the sample of studies, making it challenging to analyze the specific interventions and results in chronic diseases more precisely. Another limitation was the search strategy in electronic databases, impacting the number of studies, and the cut-off defined by the authors of chronic disease; this may affect the exclusion of other behavioral insights in the same field. As strengths of the study, we highlight the use of the PRISMA protocol and standardized instruments for the critical analysis of studies. Additionally, the interventions were from Randomized Controlled Trial studies, making it possible to indicate a causal relationship between intervention and research results, thus increasing the quality of evidence.

## 5. Conclusions

Behavioral Sciences can contribute to increased thinking about effective health interventions. Specifically, the treatment of chronic diseases can be improved with the use of behavioral insights. With these interventions, patients, healthcare teams and managers can make better decisions for the recovery and well-being of patients with chronic diseases.

Behavioral Science interventions for chronic diseases show evidence using framing messages, nudges and vouchers primarily for screening and patient adherence to treatment. The current state of decision-making primarily involves patients, with possible future studies suggesting the inclusion of healthcare professionals and decision-makers. Further research could explore behavioral science interventions and the impact on the clinic and on public health policies, focusing on improving screening tests and patient adherence to treatment.

## Figures and Tables

**Table 1:** Summary of the articles included in the final sample.

Type of Intervention in BE	Studies	Influence of Intervention on Chronic Diseases
Framing message	Alsan et al., 2020 (USA) [17]	Reduced knowledge gaps regarding COVID-19.
	Bilger et al., 2019 (Singapore) [18]	Increase in medication adherence.
	Brown et al., 2022 (USA) [19]	Increase in prostate screening in older men and overtreatment of diabetes in older adults.
	Duarte, 2021 (Chile) [20]	Increase in free mammograms.
	Gadsden et al., 2021 (Indonesia) [21]	Results for health services provided to the community for cardiovascular diseases.
	Harsin et al., 2021 (USA) [22]	Increase in intention to practice unprotected sex is associated with difficulty in making decisions in situations related to sexuality.
	Huf et al., 2020 (UK) [1]	Increased uptake of cervical cancer screening.
	Kassas and Nayga Jr., 2021 (USA) [23]	Greater adherence to COVID-19 prevention behaviors related to political party choice.
	Mehta et al., 2019 (USA) [10]	Increased active choice in colonoscopy examination.
	Omar Galárraga et al., 2018 (South Africa) [14]	Increased likelihood of condom use and dual protection.
	Roope et al., 2020 (UK) [4]	Increased positive belief in antibiotic action in influenza.
	Szilagyi et al., 2021 (USA) [15]	Not effective in raising influenza vaccination rates.
	Wagner et al., 2020 (USA) [16]	Influenced uptake of HIV testing, impacting on reduced transmission.
Messaging and incentives	Fukuma et al., 2022 (Japan) [24]	Increased the likelihood of patients’ adherence to medical appointments.
	Mehta et al., 2017 (USA) [25]	Increased colonoscopy uptake.
	Slater et al., 2017 (USA) [26]	Increased the number of mammograms received.
	Zheng et al., 2022 (China) [27]	Influential in lowering blood pressure (BP) in the short term.
Financial and mixed incentives	Asch et al., 2015 (USA) [28]	Reduced the patient’s cholesterol levels.
	McConnell et al., 2020 (USA) [29]	Influence on physicians’ behavior and patients’ medication adherence.
	Riegel et al., 2020 (USA) [2]	Increasing adherence to medicalization and decreasing readmissions.
	Zhang et al., 2022 (China) [30]	Increased adherence to treatment for eye patients.
Nudge	Hirai et al., 2016 (Japan) [31]	Increased cervical–rectal cancer (CRC) screening.
	Karim et al., 2019 (Ethiopia) [32]	Contraceptive treatment adherence.
	Krutsinger et al., 2020 (USA) [33]	Did not increase patient adherence to respiratory failure treatment.
Voucher	Bilger, Özdemir and Finkelstein, 2020 (Singapore) [9]	Increase the intention to adhere to screening for mammography.
	Weinstein et al., 2013 (Switzerland) [3]	Patients were considered more relevant in cost issues by regulators.

**Table 2:** Analysis of the EBL assessment checklist domains for the included studies.

Studies	Validity (%)				General Validity of the Study (%)
	Population	Data Collection	Study Design	Result	
Alsan et al., 2020 [17]	100	86	100	66	88
Asch et al., 2015 [28]	62	100	100	66	81
Bilger et al., 2019 [18]	62	87	100	66	77
Bilger, Özdemir and Finkelstein, 2020 [9]	55	75	80	83	71
Brown et al., 2022 [19]	75	50	60	33	56
Duarte, 2021 [20]	100	100	80	57	85
Fukuma et al., 2022 [24]	100	50	100	66	83
Gadsden et al., 2021 [21]	77	40	80	66	68
Harsin et al., 2021 [22]	22	75	100	66	61
Hirai et al., 2016 [31]	100	37	100	66	74
Huf et al., 2020 [1]	87	86	80	83	85
Kassas and Nayga Jr., 2021 [23]	75	87	80	66	78
Karim et al., 2019 [32]	87	75	100	86	87
Krutsinger et al., 2020 [33]	62	100	100	66	81
McConnell et al., 2020 [29]	100	40	100	83	83
Mehta et al., 2017 [25]	87	100	80	83	87
Mehta et al., 2019 [10]	100	75	80	83	87
Omar Galárraga et al., 2018 [14]	50	50	100	50	61
Riegel et al., 2020 [2]	11	28	60	33	30
Roope et al., 2020 [4]	55	25	80	83	59
Slater et al., 2017 [26]	55	60	60	66	60
Szilagyi et al., 2021 [15]	70	66	40	50	71
Wagner et al., 2020 [16]	87	87	100	66	85
Weinstein et al., 2013 [3]	37	87	80	66	66
Zhang et al., 2022 [30]	100	62	100	100	88
Zheng et al., 2022 [27]	55	43	100	66	63

## Data Availability

The data is available at: Corrêa, R. (2024). Tables S1 and S2.p1. Zenodo. https://doi.org/10.5281/zenodo.10938334 (accessed on 7 April 2024).

## Supplementary Materials

The following supporting information can be downloaded at: https://www.mdpi.com/article/10.3390/ijerph21070837/s1, Table S1: PRISMA 2020 Checklist; Table S2: Main findings and characteristics of studies on evidence from intervention studies that assessed the influence of Behavioral Sciences in chronic disease.
