# Supplementary material for: Assessing the Impact of Behavioral Sciences Interventions on Chronic Disease Prevention and Management: A Systematic Review of Randomized Controlled Trials
Source: Int J Environ Res Public Health. 2024 Jun 27;21(7):837. doi: 10.3390/ijerph21070837 (PMC11277013; doi:10.3390/ijerph21070837)
Supplement: Supplementary file 1 [file ijerph-21-00837-s001.zip › ijerph-2977821-supplementary.pdf]

Table S1. PRISMA 2020 Checklist

Section and Topic	Item #	Checklist item: Assessing the impact of Behavioral sciences interventions on chronic disease prevention and management: a Systematic Review of Randomized Controlled Trials	Location where item is reported
**TITLE**			
Title	1	Identify the report as a systematic review.	1
**ABSTRACT**			
Abstract	2	See the PRISMA 2020 for Abstracts checklist.	1
**INTRODUCTION**			
Rationale	3	Describe the rationale for the review in the context of existing knowledge.	1,2
Objectives	4	Provide an explicit statement of the objective(s) or question(s) the review addresses.	2
**METHODS**			
Eligibility criteria	5	Specify the inclusion and exclusion criteria for the review and how studies were grouped for the syntheses.	2
Informatio nsources	6	Specify all databases, registers, websites, organisations, reference lists and other sources searched or consulted to identify studies. Specify the date when each source was last searched or consulted.	2
Search strategy	7	Present the full search strategies for all databases, registers and websites, including any filters and limits used.	2
Selection process	8	Specify the methods used to decide whether a study met the inclusion criteria of the review, including how many reviewers screened each recordand each report retrieved, whether they worked independently, and if applicable, details of automation tools used in the process.	2
Data collection process	9	Specify the methods used to collect data from reports, including how many reviewers collected data from each report, whether they worked independently, any processes for obtaining or confirming data from study investigators, and if applicable, details of automation tools used in theprocess.	2
Data items	10a	List and define all outcomes for which data were sought. Specify whether all results that were compatible with each outcome domain in eachstudy were sought (e.g. for all measures, time points, analyses), and if not, the methods used to decide which results to collect.	2
	10b	List and define all other variables for which data were sought (e.g. participant and intervention characteristics, funding sources). Describe anyassumptions made about any missing or unclear information.	2
Study risk of bias assessment	11	Specify the methods used to assess risk of bias in the included studies, including details of the tool(s) used, how many reviewers assessed each study and whether they worked independently, and if applicable, details of automation tools used in the process.	2
Effect measures	12	Specify for each outcome the effect measure(s) (e.g. risk ratio, mean difference) used in the synthesis or presentation of results.	2
Synthesis methods	13a	Describe the processes used to decide which studies were eligible for each synthesis (e.g. tabulating the study intervention characteristics and comparing against the planned groups for each synthesis (item #5)).	2
	13b	Describe any methods required to prepare the data for presentation or synthesis, such as handling of missing summary statistics, or data conversions.	2
	13c	Describe any methods used to tabulate or visually display results of individual studies and syntheses.	2
	13d	Describe any methods used to synthesize results and provide a rationale for the choice(s). If meta-analysis was performed, describe the model(s), method(s) to identify the presence and extent of statistical heterogeneity, and software package(s) used.	2
	13e	Describe any methods used to explore possible causes of heterogeneity among study results (e.g. subgroup analysis, meta-regression).	2
	13f	Describe any sensitivity analyses conducted to assess robustness of the synthesized results.	2
Reporting bias assessment	14	Describe any methods used to assess risk of bias due to missing results in a synthesis (arising from reporting biases).	2

Certainty	15	Describe any methods used to assess certainty (or confidence) in the body of evidence for an outcome.	2

Section and Topic	Item #	Checklist item: Assessing the impact of Behavioral sciences interventions on chronic disease prevention and management: a Systematic Review of Randomized Controlled Trials	Location where item is reported
assessment			
**RESULTS**			
Study selection	16a	Describe the results of the search and selection process, from the number of records identified in the search to the number of studies included in the review, ideally using a flow diagram.	3
	16b	Cite studies that might appear to meet the inclusion criteria, but which were excluded, and explain why they were excluded.	3
Study characteristics	17	Cite each included study and present its characteristics.	3,4
Risk of bias in studies	18	Present assessments of risk of bias for each included study.	3,4
Results of individual studies	19	For all outcomes, present, for each study: (a) summary statistics for each group (where appropriate) and (b) an effect estimate and its precision (e.g. confidence/credible interval), ideally using structured tables or plots.	3,4,S2
Results of syntheses	20a	For each synthesis, briefly summarise the characteristics and risk of bias among contributing studies.	3,4
	20b	Present results of all statistical syntheses conducted. If meta-analysis was done, present for each the summary estimate and its precision (e.g. confidence/credible interval) and measures of statistical heterogeneity. If comparing groups, describe the direction of the effect.	4,S2
	20c	Present results of all investigations of possible causes of heterogeneity among study results.	4,S2
	20d	Present results of all sensitivity analyses conducted to assess the robustness of the synthesized results.	4,S2
Reporting biases	21	Present assessments of risk of bias due to missing results (arising from reporting biases) for each synthesis assessed.	4,S2
Certainty of evidence	22	Present assessments of certainty (or confidence) in the body of evidence for each outcome assessed.	3,4,S2
**DISCUSSION**			
Discussion	23a	Provide a general interpretation of the results in the context of other evidence.	4,5,6
	23b	Discuss any limitations of the evidence included in the review.	5,6
	23c	Discuss any limitations of the review processes used.	5,6
	23d	Discuss implications of the results for practice, policy, and future research.	5,6
**OTHER INFORMATION**			
Registration and protocol	24a	Provide registration information for the review, including register name and registration number, or state that the review was not registered.	6
	24b	Indicate where the review protocol can be accessed, or state that a protocol was not prepared.	6
	24c	Describe and explain any amendments to information provided at registration or in the protocol.	6
Support	25	Describe sources of financial or non-financial support for the review, and the role of the funders or sponsors in the review.	6
Competing interests	26	Declare any competing interests of review authors.	6
Availability of data, code and other materials	27	Report which of the following are publicly available and where they can be found: template data collection forms; data extracted from included studies; data used for all analyses; analytic code; any other materials used in the review.	6

PRISMA 2020 Checklist

From: Page MJ, McKenzie JE, Bossuyt PM, Boutron I, Hoffmann TC, Mulrow CD, et al. The PRISMA 2020 statement: an updated guideline for reporting systematic reviews. BMJ 2021;372:n71. doi: 10.1136/bmj.n71
For more information, visit: http://www.prisma-statement.org/

Table S2. Main findings and characteristics of studies on evidence from intervention studies that assessed the influence of Behavioral sciences in chronic disease.

Author, year, country	Study design, sample	Instruments	Intervention	Study quality	Outcome
Alsan et al., 2020 (USA)	Randomized Controlled Trail. 14,267 adults.	Knowledge and beliefs (Alsan et al., 2020).<br>Trust in the healthcare system (Alsan et al., 2020).<br>Views on mask-wearing (Alsan et al., 2020).<br>Knowledge gaps (number of errors on seven facts on COVID-19 symptoms and prevention).<br>Information-seeking behavior (number of Web links demanded out of 10 proposed).	Participants viewed 3 video messages regarding COVID-19 that varied by physician race/ethnicity, acknowledgment of racism/inequality, and community perceptions of mask-wearing.	88	The intervention demonstrated outcomes such as reducing knowledge gaps regarding Covid-19, especially in populations that generated identification with the messenger.
Asch et al., 2015 (USA)	Cluster randomized clinical trial. 340 primary care physicians (PCPs). 1,503 patients.	A web-based platform that supports recruitment, consent, randomization, and data collection for clinical trials.<br>Self-administered surveys, and transfers of financial incentives to participants.	Physician financial incentives, patient incentives, or shared physician-patient incentives.	81	Financial incentives shared between doctors and patients have reduced the patient's cholesterol levels.
Bilger et al., 2019 (Singapore)	Randomized controlled trial. 100 patients.	Adherent days at the end of the intervention period.	SIGMA (Study on Incentives for Glaucoma Medication Adherence).	77	An increase in medication adherence was observed in the participants who received discounts (vouchers).

		The monthly proportion of participants meeting the adherence target set for the intervention group to earn rebates.<br><br>Generic health status EQ-5D-5L score.<br><br>Glaucoma Quality of Life-15 (GQL-15) score.<br><br>Brief Illness Perception Questionnaire (BIPQ).<br><br>Beliefs about Medication Questionnaire (BMQ).	The intervention group was allowed to earn rebates on their treatment costs (glaucoma-related medication and check-up costs) contingent on meeting the target adherence.<br><br>The intervention and control groups received usual care, which consisted of routine check-ups with an ophthalmologist and counseling sessions with a nurse on effective glaucoma treatment, underlining the health risks raised by nonadherence to the medication regimen.		
Bilger, Özdemir & Finkelstein, 2020 (Singapore)	Randomized controlled trial. 801 patients.	Screening of mammography and Pap smear acoording the guidelines.	Esposition of half of respondents to a gain framed public health message promoting the benefits of screening.	71	Messages informing about the efficacy and cost of treatment at the time of a positive mammography test result showed the greatest influence on intention to adhere to screening.

Brown et al., 2022 (USA)	Cluster randomized controlled trial.<br>60 primary care clinics.	Electronic health record (EHR).<br>Clinical decision support (CDS).<br>EHR data documented as part of routine care delivery.<br>Electronic queries of data in the EDW copied from the EHR.	BEAGLE, CDS interventions through their EHR system plus a one-time clinician education module, or simply receive a one-time clinician education module.	56	The intervention identified prostate screening in older men and overtreatment of diabetes in older adults, increasing costs to the system.
Duarte, 2021 (Chile)	Randomized controlled trial.<br>12,000 patients.	Women who have not had mammogram for at least 24 months.	The treatment group was sent 1 mailer.<br>Women were divided into 8 equal-sized groups, 8 different types of messages and test different incentive combinations.<br>4 focus groups with 8 participants each and followed a semistructured protocol based on findings from Puschel et al. (2010).	85	The messaging intervention demonstrated outcomes such as an increase in free mammograms.
Fukuma et al., 2022 (Japan)	Randomized clinical trial.<br>4,011 patients.	Adherence to a recommended physician visit within 6 months of the intervention, identified by Medical claims data using diagnosis codes related to CKD (International classification of diseases).	Two types of interventions: (1) the nudge-based letter that contained a message on the basis of behavioral economics, (2) the clinical letter including general information about CKD risks, and (3) the control	83	The clinical and suggestion-based letters increased the likelihood of patients' adherence to medical appointments.

			(informed only of the screening results).		
Gadsden et al., 2021 (Indonesia)	Randomized clinical trial. 40 participating Kaders (community health workers), 4,084 patients and 1,153 patients in high risk.	Feasibility and acceptability of the two different forms of incentives, including their design and method of administration.	Cardio- vascular disease (CVD) (SMARThealth) (Patel, 2019).<br><br>CHWs working on a cardiovascular disease (CVD) risk screening and management programme in two villages were assigned to receive either a financial or non-financial incentive<br>Focus group discussions were conducted with CHWs and semi-structured interviews with programme administrators to investigate acceptability, facilitators and barriers to implementation and feasibility of the incentive models.	68	The intervention presented results of health services for cardiovascular diseases provided to the community.
Harsin et al., 2021 (USA)	Randomized controlled trial. 289 students.	Visual analog scale (VAS).<br><br>Sexual Desire Inventory-2 (SDI).<br><br>HIV Risk Taking Behavior Scale (HRBS).	Condom Purchase Task (CoPT).<br><br>Sexual Discounting Task, based in SDDT (Johnson and Bruner's, 2012).	61	The intention to practice unprotected sex is associated with difficulty in making decisions in situations related to sexuality (sexual discounting).
Hirai et al., 2016 (Japan)	Prospective randomized controlled trial.<br>2,140 patients.	FOBT test, community-organized colorectal cancer screening.	(1) an individual assessment, and (2) an assessment-based tailored message (frame A,B,C), based on the theory of	74	The messaging intervention increased cervical-rectal cancer (CRC) screening.

			planned behavior (Sohl & Moyer, 2007).		
Huf et al., 2020 (UK)	Randomized controlled trial.<br>Study 1: 13,133 patients.<br>Study 2: 11,405 patients.	SMS-PCP.<br><br>SMS-SNT and SMS-SNP.<br><br>SMS-GF and SMS-LF.	Based on MINDSPACE framework (Dolan et al., 2010), (6 tipes of message)	85	The behavioral SMS intervention increased uptake of cervical cancer screening.
Kassas & Nayga Jr., 2021 (USA)	Randomized controlled trial.<br>1,200 adults.	Demographic characteristics, political partisanship, income reductions due to Covid-19, smoking status, and whether or not they or someone they know (e.g., family, friend, coworker) contracted COVID-19.<br><br>General attitudes towards risk using the brief sensation seeking scale.	Messaging treatments that highlight the health risks of COVID-19. (4 tips of message: health, public health, economic risk and public health+economic risk).	78	Greater adherence to Covid-19 prevention behaviors related to political party choice
Karim et al., 2019 (Ethiopia)	Cluster randomized controlled trial.<br>660 patients.	Injectable contraceptive discontinuation rate within 12 months of uptake.<br><br>Three survey items: (1) the month and year the eli-gible participant visited the health post, (2) whether the participant was still using the injectable contraceptive without interruption at the time of the survey, (3) if the participant was not	Intervention package, consisting of a health worker planning calendar, a client counseling job aid, and client appointment cards.	87	Choice architecture influenced contraceptive treatment adherence.

		currently using the injectable contraception.			
Krutsinger et al., 2020 (USA)	Randomized controlled trials. 10 intensive care units (ICUs) 191 surrogate decision-makers (SDMs).	SDM demographic survey.<br>Nine-item comparative riskiness scale (CRS).	Ventilation weaning approaches.<br>Nudge survey based on insights from colleagues at Penn's Center for Health Incentives and Behavioral Economics.<br>Foot-in-the- door nudge (Comello et al., 2016, Girandola, 2002),	81	The intervention did not increase patient adherence to respiratory failure treatment.
McConnell et al., 2020 (USA)	Cluster randomized controlled trial.<br>90 physicians and 2,602 patients.	Measured patients' adherence using real-time data obtained for participating patients through their PBM.<br>Electronic pharmacy data capture all refills that take place at pharmacies and are linked to claims data.<br>Physicians' engagement with the medication adherence system using metadata from the study website.	Intervention components addressed to nonadherence patiente: (a) Physicians received a notification when one of their patients was nonadherent; (b) a pharmacist was made available to contact nonad-herent patients; and (c) physicians were randomized to two versions of choice architecture with regard to how they engaged the pharmacist.	83	Choice architecture had an influence on physicians' behavior and patients' medication adherence.
Mehta et al., 2017 (USA)	Randomized controlled trial. 2,245 employees.	Percentage of participants who completed colonoscopy within 3 months.	Email with the active choice (framing) to opt in or opt out of scheduling (active choice), or the active choice email plus an offer of a $100 conditional incentive to	87	The active opt-in email or together with financial incentive increased colonoscopy uptake.

			participate (financial incentive).		
Mehta et al., 2019 (USA)	Randomized controlled trial. 438 patients	CRC screening completion (FIT or colonoscopy) within 4 months of initial outreach.<br><br>CRC screening completion within 6 months of outreach and the choice of colonoscopy as a screening test.	3 outreach groups to receive mailings (framing) about CRC screening (colonoscopy only, sequential choice, active choice).	87	FIT intervention increased active choice in colonoscopy examination.
Omar Galárraga et al., 2018 (South Africa)	Randomised control pilot trial. 100 patients.	Condom use was assessed as part of a series of options for the question: "Are you currently using any method to delay or avoid getting pregnant?"<br><br>Dual protection use was assessed with the question: "Do you currently practice 'dual protection'.<br><br>Exploratory outcomes included assessing the feasibility of objectively measuring dual protection at 3 and 6 months via clinical examinations, as well as conducting urine pregnancy tests and STI.	"Empower Nudge", lottery to promote dual protection among young women seeking post-abortion care.	61	Increased likelihood of condom use and dual protection was identified in the lottery intervention.

Riegel et al., 2020 (USA)	Pilot randomized clinical trial. 130 patients.	Adherence daily and summarized it in three 30-day intervals for each person, %PDT13 monthly using the EM device.<br><br>Clinical information, and rehospitalization events were collected from the electronic medical record.	Mobile application ("app") rooted in BE created by wellth, a health-based mobile application development company, on medication adherence with call and finantial incentives.	30	The intervention had an effect on increasing adherence to medicalization and decreasing readmissions.
Roope et al., 2020 (UK)	Randomized controlled trial. 4,000 adults.	Survey abour health status: temperature, aching muscles, headache, a dry chesty cough, a sore throat, and you feel weak).	Three different messages about antibiotics and AMR, version ('fear message' empowering message)	59	The messaging intervention increased positive beliefs of antibiotic action in influenza.
Slater et al., 2017 (USA)	Randomized controlled trial. 18,939 patients.	CMS' Physician/Supplier and Outpatient Standard Analytical Files (SAF).<br><br>Completion of a screening mammogram as documented by CMS claims data in the 12-month interval after mailers were sent.	Intervention with 3 conditions: (1) Direct Mail only, (2) Direct Mail plus Incentive, and (3) Control	60	The intervention with mail plus financial incentive increased the number of mammograms received.
Szilagyi et al., 2021 (USA)	Randomized trial.<br>53 primary care practices. 196,486 patients.	Influenza Vaccination Data, electronic health records (EHRs).	Message asking if they planned on getting an influenza vaccination: (1) pre-commitment reminder alone (message), (2) pre-commitment + loss frame messages, (3) pre-commitment + gain frame	71	The messaging strategies (pre-commitment and loss/gain framing) were not effective in raising influenza vaccination rates.

			messages, (4) loss frame messages alone, (5) gain frame messages alone, or (6) standard of care control.		
Wagner et al., 2020 (USA)	Randomized controlled trial. The screening model is representative of the ZSFG ED patient population. The authors assume that the screening intervention at ZSFG will affect 7% of the population of San Francisco aged 13 through 64 years old.	The risk of undiagnosed HIV infection in the ZSFG ED population was used using methodology derived from the Denver HIV Risk Score (DRS) study.	HIV testing: opt-in and or opt-out (default choice) and finantial incentives in exchange for accepting the HIV test (Montoy, Dow, Kaplan 2018).	85	The change in choice architecture along with financial inventives influenced uptake of HIV testing impacting on reduced transmission.
Wettstein & Boes, 2021 (Switzerland)	Randomized, controlled trial. 606 adults.	The model used in this study was based on a simple CES6-function.	Online experiment, Amazon Mechanical Turk (MTurk) platform.<br><br>The reimbursement situation involved a hypothetical country with seven citizens, represented by five different types of stakeholders.<br><br>Overview decision situation, The seller offers a new treatment at a proposed price.	83	Patients were considered more relevant in cost issues by regulators.
Zhang et al., 2022 (China)	Cluster randomized, controlled trial.<br>35 rural schools, 1,390 students.	Early Treatment Diabetic Retinopathy Study charts (Precision Vision, ETDRS).	Three interventions: loss-framed group, and gain-framed group and control group (eyeglasses voucher).	88	Message framing increased adherence to treatment for eye patients.

Zheng et al., 2022 (China)	Randomized controlled trial. 217 patients.	Structured questionnaire items included demographic and lifestyle characteristics and the use of antihy- pertensive medications and concomitant drugs.<br><br>Demographic and lifestyle characteristics included sex, age, smoking, alcohol drinking, and physical activity.<br><br>Protocol from American Heart Association.	WeChat-based standard education and support (finantial incentives) for hypertension management.	63	Financial incentives were influential in lowering blood pressure (BP) in the short term.
